# First person – Andreia Nunes

**DOI:** 10.1242/dmm.049244

**Published:** 2021-08-24

## Abstract

First Person is a series of interviews with the first authors of a selection of papers published in Disease Models & Mechanisms, helping early-career researchers promote themselves alongside their papers. Andreia Nunes is first author on ‘ Identification of candidate miRNA biomarkers for facioscapulohumeral muscular dystrophy using DUX4-based mouse models’, published in DMM. Andreia is a postdoc in the lab of Peter L. Jones at the University of Nevada, Reno School of Medicine, Reno, NV, USA, investigating therapeutics for and disease mechanisms of facioscapulohumeral muscular dystrophy.


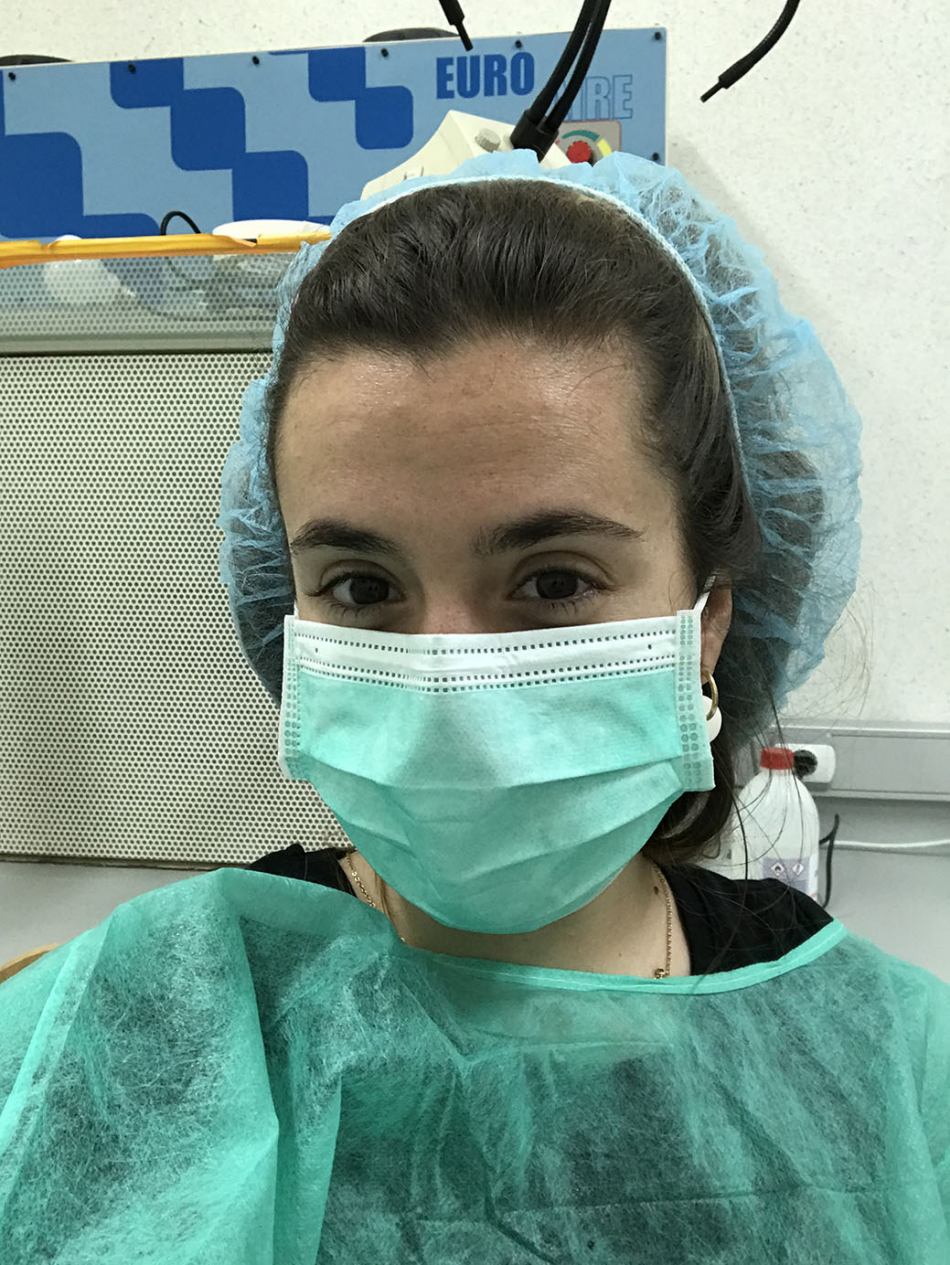


**Andreia Nunes**

**How would you explain the main findings of your paper to non-scientific family and friends?**

Blood biological markers are important measurable indicators of disease conditions, which can be used for diagnostics and to determine therapeutic efficacy. Facioscapulohumeral muscular dystrophy (FSHD) is one of the most-prevalent muscle diseases in the world, affecting males and females of all ages. Despite the major scientific progress achieved in recent years, reliable blood biological markers that correlate with disease progression and can be used for therapeutic efficacy are yet to be found for this muscle disease. In our latest study, we identified a blood biological marker whose levels are increased in blood samples from FSHD patients. We propose this blood marker as a new disease marker that can be used to monitor therapeutic efficacy in future clinical trials for FSHD.

“DUX4 expression is cytotoxic for muscle cells even when expressed at low levels.”

**What are the potential implications of these results for your field of research?**

FSHD is caused by the aberrant expression of *DUX4* in muscle cells. DUX4 expression is cytotoxic for muscle cells even when expressed at low levels. The discovery of surrogate circulating biomarkers of DUX4 expression levels are critically needed for future clinical trials testing therapeutics for this myopathy. This study identifies a new potential circulating biomarker for FSHD that might be used to determine therapeutic efficacy in future clinical trials.

**What are the main advantages and drawbacks of the model system you have used as it relates to the disease you are investigating?**

The muscles from FSHD patients are exposed to chronically low expression of double homeobox 4 (DUX4) throughout life. In our study, we demonstrate that the chronic DUX4-expressing mouse models mimic the human disease, recapitulate key aspects of human disease, and can be used to study disease mechanisms and drug and biomarker discovery. However, *DUX4* is an old-world primate gene, and the incorporation of the entire human *DUX4* gene and associated regulatory regions into the mouse genome does not necessarily completely recapitulate intrinsic regulation of *DUX4* in the human genome. Potential differences in the mouse model should be accounted for and validation with human samples is critical for reliable analyses.

“The miRNA profiling of mouse DUX4-expressing muscle samples revealed that the expression of only a few immune-related miRNAs is dysregulated in response to DUX4 expression.”

**What has surprised you the most while conducting your research?**

The miRNA profiling of mouse DUX4-expressing muscle samples revealed that the expression of only a few immune-related miRNAs is dysregulated in response to DUX4 expression. This observation suggests that DUX4 expression can trigger a very specific immune response in muscle.

**What do you think is the most significant challenge impacting your research at this time and how will this be addressed over the next 10 years?**

**Figure:**
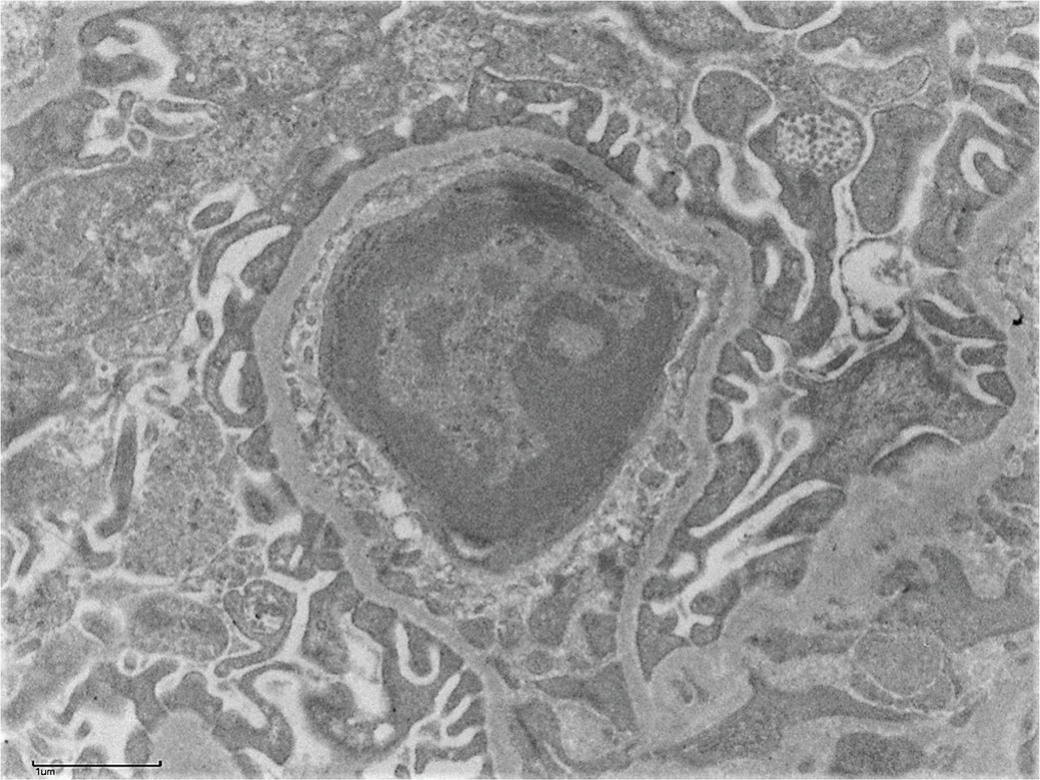
Expression of dystrophin (green) and embryonic myosin (red) in DUX4-expressing mouse muscle.

The knowledge on disease mechanisms, biomarker and therapeutic discovery for FSHD has been greatly impaired by the lack of reliable models that mimic critical aspects of the human disease. The development of mouse models for this myopathy has been a challenge until recently due to the fact that *DUX4* is an old-world primate gene and the incorporation of the entire human *DUX4* gene with its regulatory regions was technically challenging. In addition, DUX4 is highly cytotoxic even at low levels when expressed in adult somatic cells and the development of viable animals was a major drawback. As ongoing clinical trials test for new therapeutics there is still a lot to uncover about the mechanisms of this disease. A better understanding of the disease mechanisms are critical to pinpoint the best strategy to directly target DUX4 without major side effects or to target DUX4 downstream effectors. As new and more reliable mouse models have been recently developed, I think there will be progress on the knowledge on the mechanisms involved in the disease pathology in the upcoming years.

**What changes do you think could improve the professional lives of early-career scientists?**

Funding opportunities are a critical aspect of the professional lives of early-career scientists. I think the most important change that can be implemented is the creation of additional specific funding opportunities for young scientists.

**What's next for you?**

I will continue to pursue an independent research position in academia as I am enthusiastic about research and I have an ambition to develop new therapies for FSHD.
